# Supplementary material for: Generating New Coordination Compounds via Multireference Simulations, Genetic Algorithms, and Machine Learning: The Case of Co(II) and Dy(III) Molecular Magnets
Source: JACS Au. 2025 Jul 29;5(8):3808–21. doi: 10.1021/jacsau.5c00502 (PMC12381707; doi:10.1021/jacsau.5c00502)
Supplement: Supplementary file 1 [file au5c00502_si_001.pdf]

Supplementary Information

Generating new coordination compounds via multireference simulations, genetic algorithms and machine learning: the case of Co(II) and Dy(III) molecular magnets

Lion Frangoulis, Zahra Khatibi, Lorenzo A. Mariano, and Alessandro Lunghi*

School of Physics, AMBER and CRANN Institute, Trinity College, Dublin 2, Ireland

* lunghia@tcd.ie

RANDOM FOREST ADDITIONAL TESTS

To further verify the reliability of the RF model, we first show the precision, accuracy and recall for both the compounds found in COMPASS and in the static GA studies, ranked by their anisotropy, in Figure S2 and S1. For the COMPASS dataset, there is a clear trend for higher Recall on high D compounds, signifying that the RF model is less likely to discard high-interest compounds. The same result is not reproduced on the compounds identified by the GA, where the Recall is relatively independent of the location of the compounds. Verifying the exact impact of the RF model on the dynamic GA would require multiple independent runs of the algorithm within the same parameters, half with and half without the model guiding their progression, to gather statistically significant results. We instead perform the same benchmarking as in Figure 2 of the main manuscript, but now compare the performance of the static GA with both random search and a static GA accelerated by the RF model. Figure S3 of the main manuscript shows the growth of RF-guided and non-RF-guided GA versus random search simulations, averaged over a thousand runs with a termination criterion of finding one of the top ten compounds. The RF model marks only one third of the COMPASS dataset as class 1, therefor is expected to show a significant speedup of the GA+RF model compared to pure GA and random search, as can be seen in their convergence. This requires a relaxed termination criteria to be properly usable, for the small chance of discarding the top compound would lead to failed convergence for requiring to find the very best compound in a different dataset.

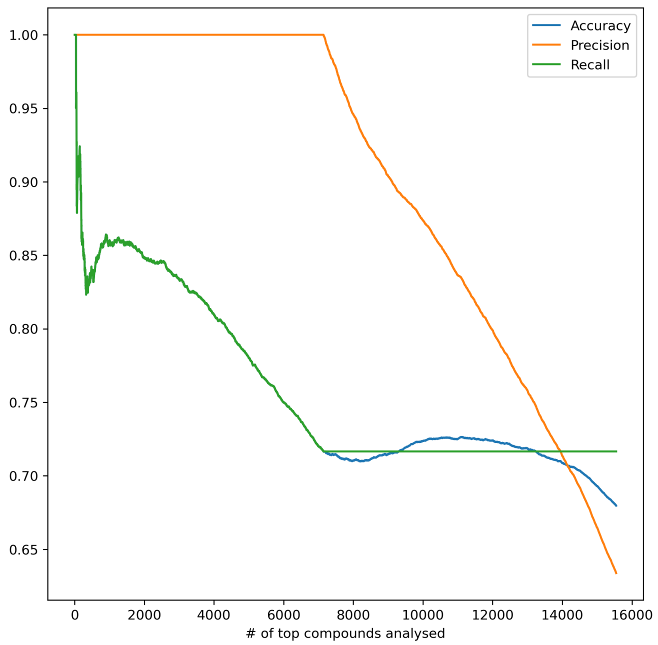

FIG. S1: Accuracy, Precision and Recall calculated for subsets of all compounds found in COMPASS, ranked by their magnetic anisotropy D. This shows that on compounds with higher D, the recall is significantly better than on lower ranked compound, meaning the RF model is less likely to discard high D compounds.

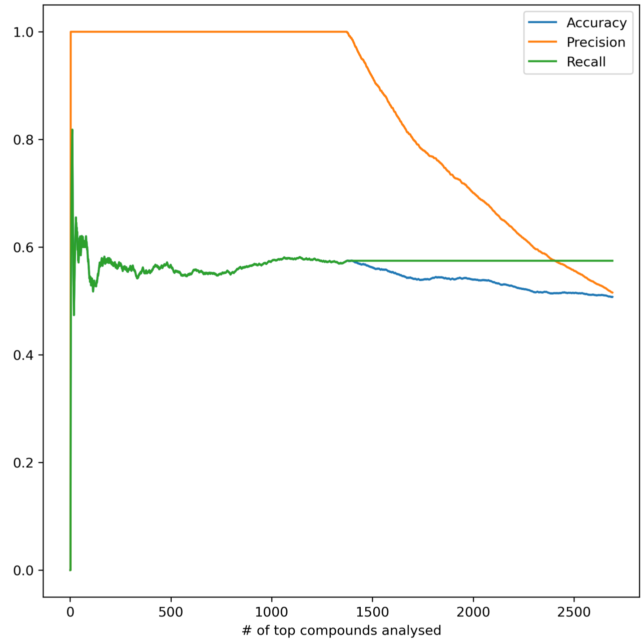

FIG. S2: Accuracy, Precision and Recall calculated for subsets of all compounds found in the static GA study, ranked by their magnetic anisotropy D. In this case, there is no correlation visible between D and the recall probability.

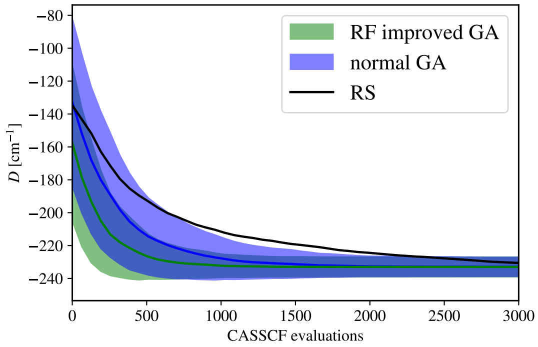

FIG. S3: Statistical growth of the minimum magnetic anisotropy D for a random search within COMPASS, and simulated runs of GA with and without RF acceleration, averaged over a thousand simulations each and a termination criteria of finding one of the top ten compounds.

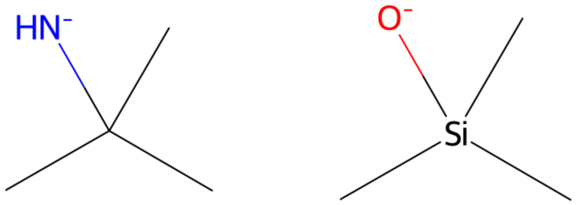

FIG. S4: **Fixed ligands for Co's dynamic GA runs.** Molecular graphs of the two static ligands selected for Co's dynamic GA runs, chosen from the most frequently occurring ligands among the top 100 high-performing compounds present in COMPASS.

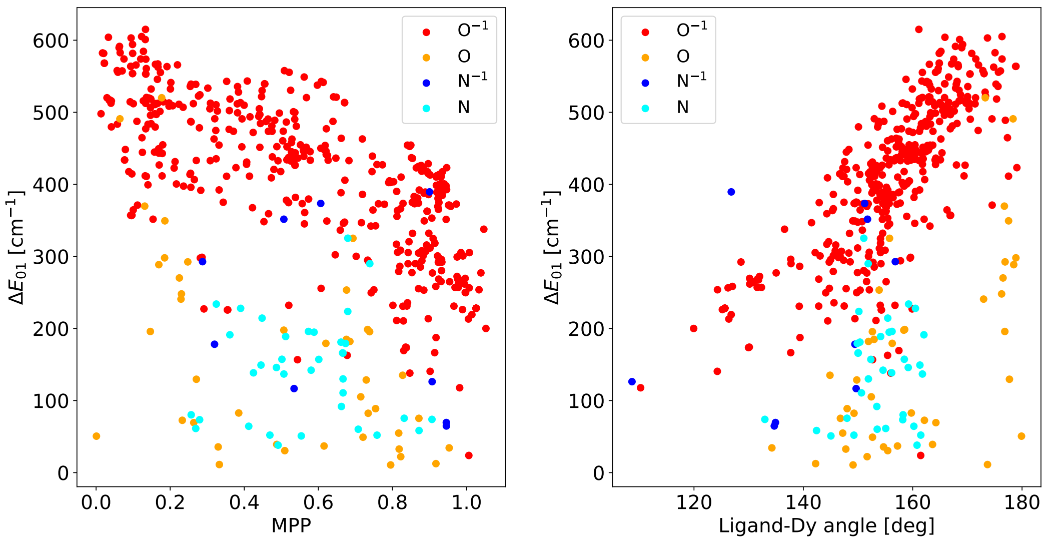

FIG. S5: Left: Correlation between the first Kramers Gap E_{01} and the molecular planarity parameter calculated on the planar ligands' connecting atoms. Right: Correlation between the first Kramers Gap E_{01} and the angle between the bond of the central atom and the axial ligands' connecting atoms.

MAGNETO-STRUCTURAL CORRELATIONS IN DY COMPOUNDS

To further understand the behavior of ΔE_{01}, we show its correlation to both the molecular planarity score and the angle between the ligands connecting atom and the Dy core in Figure S5 for all four available connecting atom types. It quickly becomes clear again that O^{-} is capable of producing the best compounds, with the ideal compound having perfectly planar water molecules in plane, as measured by their MPP [1], and a 180° angle between the axial ligands, enforcing the maximum symmetry in this direction. The behavior of ΔE_{01} can mostly be explained by just these parameters and the bond distance of the axial ligands, as shown in the neural network analysis in Figure 9 of the main manuscript, with the second coordination shell playing only a minor role.

FORMATION ENERGY CALCULATION

To assess the chemical feasibility of the generated compounds, we compute the formation energies of the best-performing 192 compounds selected from different GA cycles. Previous benchmark studies on the thermochemistry of open-shell transition-metal compounds highlight the good performance of the TPSS0-D3(BJ) functional[2], which is found among the best-performing methods on the fourth rung of Jacob's ladder in DFT [3]. TPSS0-D3(BJ) is a hybrid meta-GGA density functional that combines the TPSS (Tao–Perdew–Staroverov–Scuseria)[4] meta-GGA functional with 25% Hartree–Fock exchange, and includes empirical dispersion corrections via the D3(BJ) scheme (Grimme's D3 dispersion with Becke–Johnson damping)[5]. For comparison, we also test the BP86-D3(BJ) functional, as it has been used throughout this study for the geometric optimization of the structures. For each structure, the formation energy E_f is computed using:

$$E_\mathrm{f} = E_\mathrm{comp} - \sum_i E_i^\mathrm{lig} - E_\mathrm{Co} \;, \tag{1}$$

where E_comp is the total energy of the compound, E^lig the energy of each ligand, and E_Co the energy of the isolated Co(II) ion. Both the compounds and the ligands were reoptimized consistently employing the same DFT functional. The results are reported in Fig. S6. For comparison, we also evaluate the formation energy of the single-molecule magnet $[\mathrm{Co(C_3S_5)_2}]^{2-}$ [6] adopting the same strategy. By using TPSS0-D3(BJ), we calculate $E_\mathrm{f} = -1.228564$ $\mathrm{E_h}$, while $E_\mathrm{f} = -1.271467$ $\mathrm{E_h}$ is obtained at the BP86-D3(BJ) level of theory. Results indicate that all compounds explored by the GA have a negative formation energy, comparable in magnitude to $[\mathrm{Co(C_3S_5)_2}]^{2-}$, and are therefore predicted to be thermodynamically stable according to this simple calculation.

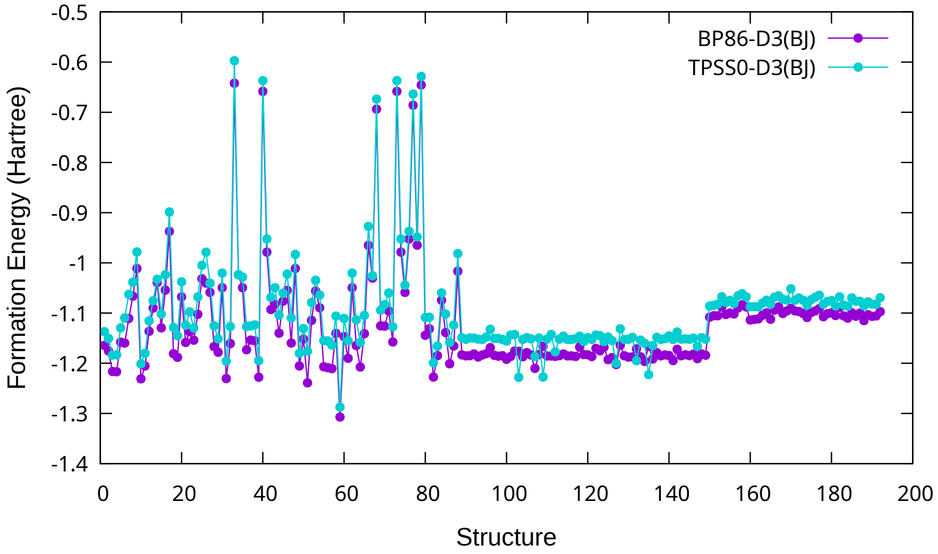

FIG. S6: Computed formation energy (Hartree) for 192 selected structures. Computed values using BP86-D3(BJ) and TPSS0-D3(BJ) DFT functionals are reported.

References

[1] Tian Lu, "Two simple and reliable metrics of molecular planarity: Molecular planarity parameter (mpp) and span of deviation from plane (sdp)," (2021), 10.26434/chemrxiv.14740344.v1.

[2] Stefan Grimme, "Accurate calculation of the heats of formation for large main group compounds with spin-component scaled mp2 methods," The Journal of Physical Chemistry A **109**, 3067–3077 (2005).

[3] Leonard R Maurer, Markus Bursch, Stefan Grimme, and Andreas Hansen, "Assessing density functional theory for chemically relevant open-shell transition metal reactions," Journal of Chemical Theory and Computation **17**, 6134–6151 (2021).

[4] Jianmin Tao, John P Perdew, Viktor N Staroverov, and Gustavo E Scuseria, "Climbing the density functional ladder: Nonempirical meta–generalized gradient approximation designed for molecules and solids," Physical review letters **91**, 146401 (2003).

[5] Stefan Grimme, Stephan Ehrlich, and Lars Goerigk, "Effect of the damping function in dispersion corrected density functional theory," Journal of computational chemistry **32**, 1456–1465 (2011).

[6] Majed S Fataftah, Joseph M Zadrozny, Dylan M Rogers, and Danna E Freedman, "A mononuclear transition metal single-molecule magnet in a nuclear spin-free ligand environment," Inorganic chemistry **53**, 10716–10721 (2014).
